# Evolocumab on top of empagliflozin improves endothelial function of individuals with diabetes: randomized active-controlled trial

**DOI:** 10.1186/s12933-022-01584-8

**Published:** 2022-08-06

**Authors:** Andrei C. Sposito, Ikaro Breder, Joaquim Barreto, Jessica Breder, Isabella Bonilha, Marcus Lima, Alessandra Oliveira, Vaneza Wolf, Beatriz Luchiari, Helison R. do Carmo, Daniel Munhoz, Daniela Oliveira, Otavio R. Coelho-Filho, Otavio R. Coelho, Jose Roberto Matos-Souza, Filipe A. Moura, Luiz Sergio F. de Carvalho, Wilson Nadruz, Thiago Quinaglia, Sheila T. Kimura-Medorima, the EXCEED-BHS3 Group

**Affiliations:** 1 Division of Cardiology, State University of Campinas (Unicamp), Campinas, Sao Paulo, 13084-971 Brazil; 2 Division of Cardiovascular Medicine, Brigham and Women’s Hospital, Harvard Medical School, Boston, USA; 3 Brazilian Heart Study Group, State University of Campinas (Unicamp), Campinas, Sao Paulo, 13084-971 Brazil

**Keywords:** Endothelial dysfunction, PCSK9i, Flow-mediated dilation

## Abstract

**Background:**

Sodium-glucose cotransporter 2 inhibitors (SGLT2i) improve endothelial dysfunction and reduce cardiovascular events in individuals with type 2 diabetes (T2D). Proprotein convertase subtilisin/kexin 9 (PCSK9i) inhibitors reduce cardiovascular events in high-risk patients. Whether the addition of PCSK9i to SGLT2i treatment adds benefits is not known.

**Objectives:**

To assess the PCSK9-i effect on the endothelial function of T2D individuals under treatment with SGLT2-i.

**Methods:**

Individuals with T2D were randomized in a 1:1 ratio to a 16-week treatment with either empagliflozin (E) or empagliflozin plus evolocumab (EE). The primary endpoint was post-treatment change from baseline in flow-mediated dilation (FMD) at 1-min. Secondary outcomes included changes in plasma levels of nitric oxide metabolites and isoprostane.

**Results:**

A total of 110 patients were enrolled, the mean age was 58 years, and 71% were men. The median post-treatment change in FMD at 1-min was 2.7% (interquartile range [IQR]: 0.9%) and 0.4% (IQR: 0.9%) in the EE and E groups, respectively (p < 0.001). There was a greater increase in plasma levels of nitrate [5.9 (16.5) vs. 2.6 (11.8); p = 0.001] and nitrite [0.14 (0.72) vs. 0.02 (0.74); p = 0.025] in the EE group than in the E group, respectively. Isoprostane reduction was more pronounced in the EE group when compared to the E group [−1.7 (5.9) vs. −1.1 (5.3); p < 0.001).

**Conclusions:**

In individuals with T2D, the addition of evolocumab on top of empagliflozin improves endothelial function.

**Supplementary Information:**

The online version contains supplementary material available at 10.1186/s12933-022-01584-8.

## Introduction

Newer pharmacological agents for type 2 diabetes mellitus (T2D), such as glucagon-like peptide 1 agonists (GLP-1a) and sodium-glucose transporter-2 inhibitors (SGLT2i), have demonstrated considerable benefit against atherosclerotic cardiovascular events [1]. Nevertheless, despite its effect, individuals with T2D remain at a high risk of developing cardiovascular outcomes. Proprotein convertase subtilisin/kexin 9 (PCSK9-i) inhibitors have demonstrated similar efficacy in reducing cardiovascular events [2, 3]. The trials that tested SGLT2i and PCSK9-i have essentially occurred in parallel and FDA approval dates are within relative proximity for each of the drugs. Although the combined use of both drug classes is likely additive in mitigating cardiovascular risk, there are no data available to support this hypothesis.

Endothelial dysfunction is an early manifestation of both atherosclerotic disease and T2D, and its attenuation may occur early after the initiation of therapies bearing an anti-atherosclerotic effect [4–6]. Flow-mediated dilation (FMD) of the brachial artery one minute after cuff deflation has become the standard parameter to quantify endothelial function and is a useful surrogate endpoint because of its noninvasiveness, its close correlation with coronary endothelial function, and its association with long-term incidence of coronary events [7, 8]. A positive effect of SGLT2i on endothelial function, as assessed by FMD, has been demonstrated by us and others in patients with T2D [9, 10]. In patients with HIV, dyslipidemia, or recent myocardial infarction, treatment with evolocumab has improved endothelial function [11, 12]. In this context, our goal was to evaluate the effect of the combination of both therapies on the endothelial function of patients with T2D.

## Methods

### Study design

The EXpanded Combination of Evolocumab plus Empagliflozin on Diabetes Trial (EXCEED- BHS3 Trial) was a randomized, prospective, single-center, open-label clinical trial. Blinding of the study for the primary endpoint was implemented by performing offline assessments of the video recordings generated during the FMD examination, as described below. The study protocol has been previously described in detail elsewhere [13]. Briefly, individuals with T2D, aged 40–70 years, with a body mass index (BMI) < 40 kg/m2, were summoned by social media and newspaper campaigns to participate in the screening visit for medical evaluation, biochemical analysis, and screening for FMD. Exclusion criteria included renal dysfunction (glomerular filtration rate < 60 ml/min/m^2^), heart failure with reduced ejection fraction (< 50%), any major cardiovascular event (such as myocardial infarction or stroke) occurring less than 6 months before enrolment, alcohol abuse, and use of SGLT2i, thiazolidinediones, or GLP-1a within 4 months before randomization. The study was conducted according to the principles of Good Clinical Practice, to the Declaration of Helsinki and it was approved by the institutional ethical committee (CAAE: 88800718.0.0000.5404). The study was registered at clinicaltrials.gov (NCT03932721) and the report of the study was oriented by the CONSORT guidelines.

Eligible participants were enrolled in a 16-week run-in phase aimed at targeting the following requirements: (i) HbA1c between 7 and 9%; (ii) low-density lipoprotein cholesterol (LDL-C) 70–100 mg/dL using the maximally tolerated dose of rosuvastatin or simvastatin; (iii) blood pressure (BP) < 140/90 mmHg; and (iv) screening FMD at 1-min between 1 and 12%. Adjustment of therapies was performed in the first 4 weeks of the run-in and in the following 12 weeks, the first 110 patients who achieved regular control of blood pressure, LDL-C, and HbA1c were selected. After the initial adjustment of medications, no changes in therapeutic regimens were made until the end of the trial. Participants fulfilling these criteria were randomly assigned (1:1) to a 16-week treatment with either empagliflozin (25 mg/day) or empagliflozin (25 mg/day) plus evolocumab (140 mg every 2 weeks). During treatment, blood samples were collected for biochemical assessments, FMD, and BP measurements were performed before dose administration at the randomization visit and again in the eighth and 16th week of treatment. Additional file 1: Figure S1 depicts the study design.

### Flow-mediated dilation

Briefly, trained examiners assessed FMD with a 5–13 MHz linear array transducer (Epiq CVX, Philips, Eindhoven, the Netherlands) sustained in an upright position with a probe holder (Quipu, Pisa, Italy). The probe was positioned to obtain a regular longitudinal brachial artery image with clear vascular boundaries. Real-time 2D images and simultaneous pulsed-wave Doppler flow of the brachial artery were continuously recorded using a validated video capture device (Epiphan’s DVI2USB 3.0, Epiphan Video, Ottawa, Canada) connected to a dedicated computer. Exam preparation included a 48-h withdrawal of medicines with significant direct vascular effects, 24-h caffeine abstinence, and 12-h fasting, according to FMD guidelines [14]. Diameter and flow changes were provoked by inflation of an appropriately sized BP cuff placed in the forearm up to 50 mmHg above the systolic BP for 5 min and then deflated. Brachial artery diameter was continuously recorded for 1 min at baseline (before inflation), 5 min of ischemia (during inflation), hyperemia (1st minute after cuff deflation), and 5 min in the dilation period. Fifteen minutes after the first (baseline) FMD, reperfusion injury of the brachial artery was induced by cuff-induced ischemia (50 mmHg above the systolic BP for 15 min followed by 15 min of reperfusion. The second FMD was obtained post-ischemia and reperfusion (post-I/R) and the aforementioned parameters were reassessed. Additional file 1: Figure S2 shows the FMD procedure. The video was processed by blinded observers using an offline automatic edge-detecting software (LabVIEW 6.02, National Instruments). The main result of the FMD is presented as the mean change (%) in diameter, calculated as [(median dilation diameter—median basal diameter)/median basal diameter]. The intersession coefficient of variability of the software for FMD measurement in the same video record analyzed by 10 different observers was 0.26%.

### Blood pressure

Office BP measurements were performed using the HEM-7113 Omron Healthcare (São Paulo, Brazil) device, according to the latest guidelines [15]. After 3 min of rest, three consecutive measurements were obtained with the patient sitting and then standing, and the mean value of the last two measurements for each position was considered. A 24-h ambulatory BP monitoring (ABPM) was obtained within one week before randomization and up to one week after the 16-week treatment period (SpaceLabs, model 90207-8Q, USA).

### Biochemical analysis

At the baseline and the 16th week of therapy, after 12-h fasting, peripheral blood samples were collected according to the guidelines [16], centrifuged at 3500 rpm, and analyzed for the following measurements: HbA1c, fasting blood glucose, aspartate aminotransferase (AST), alanine aminotransferase (ALT), urea and creatinine, high-sensitive C-reactive protein (CRP) (Cobas c702 Roche, Germany). Plasma-EDTA samples were also employed in the continuous density gradient ultracentrifugation technique to isolate very-low-density lipoprotein (VLDL), LDL, and high-density lipoprotein (HDL) fractions for total cholesterol measurement using enzymatic methods. The glomerular filtration rate was calculated using the Chronic Kidney Disease Epidemiology Collaboration (CKD-EPI) equation. Plasma aliquots were stored to measure inflammatory markers such as soluble vascular cell adhesion molecule 1 (sVCAM-1) (Invitrogen™ eBioscience™ ProcartaPlex Human VCAM-1 Simplex kit, USA) and isoprostane (Free 8-Isoprostane ELISA Kit, Cayman Chemical Company, USA). Plasma levels of nitrate and nitrite were measured using nitric oxide (NO) chemiluminescence analyzer (Model NOA, Sievers Instruments, Boulder, CO, USA).

### COVID19

On March 20, 2020, the Brazilian government declared a state of public calamity owing to the Covid19 outbreak. In line with this, between March 2020 and the study termination in October 2020, all participants were tested for Covid19 using both an RT-PCR nasopharyngeal sampling qualitative assay and a serological test within 48 h before FMD exams. Moreover, all randomized individuals were contacted weekly by the study investigators for symptom assessment.

### Endpoints

The primary endpoint was the change in FMD at 1-min after 16 weeks of therapy. FMD at 1-min after 8 weeks of therapy and the change in post-I/R FMD at 1-min after 8 and 16 weeks of therapy were the secondary endpoints. Other secondary endpoints were the change at 8 and 16 weeks of treatment in the area under the curve (AUC) of FMD during the 5-min period after cuff deflation, peak systolic velocity (PSV), end-diastolic velocity (EDV), and plasma levels of nitrate, nitrite, isoprostane, and sVCAM1.

### Statistical analysis

Data are presented as mean ± standard deviation (SD) when normally distributed or median and interquartile range (IQR) when nonparametric. Kolmogorov–Smirnov test was used to check normality. Continuous variables were compared between groups using the Student’s t-test or Wilcoxon-Mann–Whitney U test. Due to the non-normal distribution, changes in all endpoints were ranked to suit normal distribution and compared between groups by RANCOVA using baseline values as the covariate. Statistical significance was set at P < 0.05. SPSS 25. for Mac was used in all the analyses.

## Results

### Study population

Of the 1041 individuals assessed for eligibility, 246 entered the run-in phase, and the first 110 participants who met the inclusion criteria were randomized 1:1 to either empagliflozin alone (E) or evolocumab plus empagliflozin (EE). There was no dropout and all participants performed all study assessments. The study flow diagram is shown in Supplemental Material (Additional file 1: Fig S3). The study individuals had a mean age of 58 years, 71% were male, and T2D duration averaged 10 years. Except for lower pretreatment HDL-C values in the E group, baseline characteristics were comparable between the treatment arms and are summarized in Table 1. The absolute values of each clinical and laboratory parameter are listed in Additional file 1: Table S1. At randomization, all patients were taking the maximally tolerated dose of statins. As commented above, no changes were made to any of the drug therapies, including the dose of statins, during the study. The therapies at the moment of randomization are presented in Additional file 1: Table S2.

**Table 1:** Characteristics of the study population at randomization

Variable	EE	E	*p-value*
n	55	55	
Age, years	58 ± 6	58 ± 8	*0.580*
Gender male, %	73	69	*0.821*
T2D duration, years	10 (10)	9 (10)	*0.181*
CVD, %	6 (11)	3 (5.8)	*0.841*
Hypertension, %	67	76	*0.538*
Prior smoking habit, %	45	38	*0.556*
Sedentarism, %	38	36	*0.876*
Statin use, %	100	100	*1.000*
Heart rate, bpm	72 ± 10	72 ± 10	*0.860*
Office SBP, mmHg	135 ± 15	134 ± 14	*0.783*
Office DBP, mmHg	82 ± 8.4	81 ± 10	*0.884*
24 h SBP, mmHg	124 ± 16	127 ± 6.1	*0.937*
24 h DBP, mmHg	74 ± 8.7	72 ± 0.9	*0.650*
Body weight, Kg	87 ± 17	87 ± 15	*0.902*
Height, m	1.7 ± 0.1	1.7 ± 0.1	*0.423*
Body Mass Index, Kg/m^2^	31 ± 5.1	31 ± 4.2	*0.800*
Waist circumference, cm	105 ± 14	106 ± 15	*0.343*
*Biochemical analysis*			
Hemoglobin, g/dL	14 ± 1.2	14 ± 1.2	*0.301*
Fasting Blood Glucose, mg/dL	172 (60)	167 (58)	*0.917*
HbA1c, %	7.8 (1.2)	8.1 (0.9)	*0.583*
LDL-C, mg/dL	84 ± 13	83 ± 13	*0.915*
HDL-C, mg/dL	41 (12)	36 (12)	*0.037*
VLDL-C, mg/dL	33 (22)	32 (20)	*0.592*
Triglycerides, mg/dL	166 (110)	163 (108)	*0.507*
Creatinine, mg/dL	0.9 (0.3)	0.8 (0.2)	*0.205*
Urea	32 (14)	32 (14)	*0.822*
C-Reactive Protein, mg/dL	0.2 (0.3)	0.2 (0.4)	*0.706*
AST, U/L	18 (7)	19 (9)	*0.480*
ALT, U/L	23 (11)	25 (18)	*0.254*
*FMD, mm*			
1-min	2.50 (1.64)	2.70 (2.16)	*0.156*
2-min	1.75 (1.65)	1.83 (2.22)	*0.797*
3-min	1.12 (2.38)	1.07 (2.08)	*0.988*
4-min	0.37 (1.21)	0.26 (1.10)	*0.720*
5-min	0.00 (0.25)	0.00 (0.14)	*0.468*
AUC	3.87 (5.620	5.08 (5.87)	*0.862*
*Blood velocity parameters*			
PSV			
0-min	73.35 (27.70)	78.43 (30.80)	*0.879*
1-min	79.44 (28.90)	81.98 (31.20)	*0.988*
5-min	72.75 (27.50)	73.57 (24.20)	*0.940*
EDV			
0-min	4.75 (3.20)	4.34 (5.40)	*0.556*
1-min	6.39 (4.90)	5.36 (4.90)	*0.593*
5-min	3.36 (3.50)	3.97 (4.00)	*0.517*
*Plasma biomarkers of endothelial function*			
Nitrate			
0-min	26.70 (13.20)	27.60 (13.40)	*0.085*
1-min	27.30 (12.60)	27.00 (11.10)	*0.505*
5-min	26.40 (12.00)	25.70 (11.10)	*0.393*
Nitrite			
0-min	0.65 (1.84)	0.65 (1.64)	*0.722*
1-min	0.84 (1.60)	0.78 (1.74)	*0.820*
5-min	0.81 (1.43)	0.91 (1.47)	*0.582*
*Plasma biomarkers of vascular inflammation*			
Isoprostane, pg/mL	15.65 (6.13)	15.22 (5.41)	*0.176*
*sVCAM1, ng/mL*	411.65 (0.60)	411.80 (0.90)	*0.709*

T2D, type 2 diabetes mellitus; CVD, cardiovascular disease; SBP, systolic blood pressure; DBP, diastolic blood pressure; HbA1c, glycosylated hemoglobin; LDL-C, low-density lipoprotein cholesterol; HDL-C, high-density lipoprotein cholesterol; VLDL, very low-density lipoprotein cholesterol; AST, aspartate transaminase; ALT, alanine transaminase; FMD, flow-mediated dilation; PSV, peak systolic velocity; EDV, end-diastolic velocity; sVCAM1, soluble vascular cell adhesion molecule 1

At 8 weeks after randomization, systolic BP was equally reduced by approximately 6 mmHg in both EE and E groups, respectively (p = 0.781). The median reduction in diastolic BP was 5 mmHg in the EE arm, and 2 mmHg in the E group (p = 0.726). Study arms had similar reductions in the body mass index and HbA1c levels, but fasting blood glucose had a greater median reduction in E than in the EE arm (38 mg/dL vs. 40 mg/dL; p = 0.011). As expected, compared with the E arm, the EE group showed larger reductions in LDL-C levels (Table 2).

**Table 2:** Posttreatment changes

	Time-point					
	8 weeks			16 weeks		
Variable	EE	E	p-value	EE	E	p value
Heart rate, bpm	6.0 (15)	10 (17)	0.075	1.0 (14)	0.0 (7)	*0.002*
Office SBP, mmHg	−6 (15)	−6 (17)	*0.781*	−10 (18)	−4 (20)	*0.284*
Office DBP, mmHg	−5 (11)	−2 (9)	*0.726*	−4 (11)	−2 (13)	*0.197*
Body weight, Kg	−1.8 (2.1)	−1.5 (2.2)	*0.935*	−2.3 (3.1)	−2.2 (3.1)	*0.788*
Body Mass Index, Kg/m^2^	−0.7 (0.7)	−0. 6 (1.0)	*0.905*	−0. 8 (1.0)	−0.7 (1.3)	*0.391*
Waist circumference, cm	−0.7 (0.7)	−0. 6 (1.0)	*0.989*	−1.3 ± 3.1	−1.6 ± 3.5	*0.983*
Biochemical analysis						
Hemoglobin, g/dL	0.7 (1.1)	0.5 (1.3)	0.026	0.8 (0.9)	0.75 (1.4)	*0.233*
Fasting Blood Glucose, mg/dL	−38 (43)	−44 (40)	*0.011*	−38 (48)	−41 (44.5)	*0.089*
HbA1c, %	−0.8 (0.8)	−1.0 (0.8)	*0.609*	−0.7 (1.0)	−0.9 (0.9)	*0.824*
LDL-C, mg/dL	−41 ± 19	−5 ± 20	< *0.001*	−44 ± 18	−1.1 ± 16	< *0.001*
HDL-C, mg/dL	0.0 (7)	0.9 (10)	*0.806*	−2.0 (9)	2.14 (9)	*0.515*
VLDL-C, mg/dL	−7 (12)	−3 (12)	*0.184*	−8 (12)	−3 (15)	*0.038*
Triglycerides, mg/dL	−31 (54)	−19 (67)	*0.315*	−39 ± 52	−28 ± 69	*0.132*
Creatinine, mg/dL	0.00 (0.14)	0.03 (0.13)	*0.105*	−0.03 (0.13)	0.03 (0.12)	*0.222*
C-Reactive Protein, mg/dL	0.02 (0.11)	0.00 (0.15)	*0.172*	0.01 (0.17)	0.01 (0.20)	*0.957*
AST, U/L	0.00 (5.00)	0.00 (5.50)	0.014	1.0 (5.25)	1.00 (5.00)	*0.003*
ALT, U/L	0.50 (6.25)	1.00 (11.00)	0.001	3.00 (6.00)	4.00 (9.00)	*0.001*
Urea, mg/dL	4.00 (11.00)	4.00 (8.00)	0.010	3.00 (12.00)	3.00 (10.00)	*0.010*
FMD, mm						
1-min	1.97 (0.54)	0.33 (0.25)	< *0.001*	2.72 (0.93)	0.41 (0.88)	< *0.001*
2-min	1.64 (0.45)	0.34 (0.61)	< *0.001*	2.24 (0.96)	0.32 (0.77)	< *0.001*
3-min	1.06 (0.93)	0.10 (0.78)	< *0.001*	1.73 (1.37)	0.31 (1.06)	< *0.001*
4-min	0.81 (1.15)	0.39 (1.17)	< *0.001*	1.18 (1.41)	0.56 (1.10)	< *0.001*
5-min	0.29 (1.26)	0.19 (0.73)	< *0.001*	0.98 (1.37)	0.44 (0.92)	< *0.001*
AUC	10.04 (4.60)	6.18 (4.92)	< *0.001*	12.12 (3.98)	7.28 (4.39)	< *0.001*
Blood velocity parameters						
PVS, cm/sec						
0-min	4.4 (5.6)	1.9 (4.1)	< *0.001*	15.2 (17.6)	4.1 (13.4)	*0.002*
1-min	0.7 (9.9)	0.1 (5.0)	*0.524*	23.3 (18.4)	11.2 (12.7)	*0.001*
5-min	5.6 (11.1)	4.6 (9.4)	*0.484*	22.1 (19.3)	11.2 (14.9)	*0.019*
EDV, cm/sec						
0-min	0.05 (0.03)	-0.45 (0.06)	< *0.001*	0.19 (0.13)	0.87 (0.11)	< *0.001*
1-min	0.38 (0.34)	0.05 (0.05)	< *0.001*	0.60 (0.56)	0.16 (0.17)	< *0.001*
5-min	0.36 (0.35)	0.12 (0.13)	< *0.001*	0.06 (1.59)	0.00 (1.65)	< *0.001*
Plasma biomarkers of endothelial function						
Nitrate, nmol/L						
0-min	1.40 (10.90)	1.30 (10.52)	*0.056*	5.90 (16.50)	2.60 (11.80)	*0.001*
1-min	4.13 (16.60)	2.45 (12.50)	*0.030*	9.20 (19.30)	3.90 (12.30)	*0.007*
5-min	3.20 (15.98)	1.53 (11.41)	*0.006*	6.40 (17.80)	1.50 (12.15)	*0.007*
Nitrite, nmol/L						
0-min	0.18 (0.53)	0.06 (0.63)	*0.012*	0.14 (0.72)	0.02 (0.74)	*0.025*
1-min	0.14 (0.85)	0.03 (0.71)	*0.009*	0.22 (1.12)	0.02 (0.81)	*0.058*
5-min	0.02 (0.57)	0.00 (0.61)	*0.082*	0.12 (0.76)	0.07 (0.47)	*0.010*
Plasma biomarkers of vascular inflammation						
Isoprostane, pg/mL	na	na	na	−1.68 (5.87)	−1.12 (5.34)	< *0.001*
sVCAM1, ng/mL	na	na	na	−0.20 (0.85)	−0.32 (1.16)	*0.524*
24 h BP Monitoring						
SBP, mmHg	na	na	na	−8.0 (13)	−4 (16)	*0.363*
DBP, mmHg	na	na	na	−3.0 (7.5)	−3.5 (11.25)	*0.419*

SBP systolic blood pressure, DBP diastolic blood pressure, HbA1c glycosylated hemoglobin, LDL-C, low-density lipoprotein cholesterol; HDL-C, high-density lipoprotein cholesterol; VLDL, very-low-density lipoprotein cholesterol; AST, aspartate transaminase; ALT, alanine transaminase; FMD, flow-mediated dilation; PSV, peak systolic velocity; EDV, end-diastolic velocity; sVCAM1, soluble vascular cell adhesion molecule 1; na, not available

After 16 weeks, changes in office systolic BP, diastolic BP, and 24-h systolic and diastolic BP were equivalent between groups (Table 2). There was a similar decrease in BMI in both groups. The median reduction in HbA1c and fasting blood glucose were equivalent between groups (Table 2). 

### FMD change

**Fig. 1:**
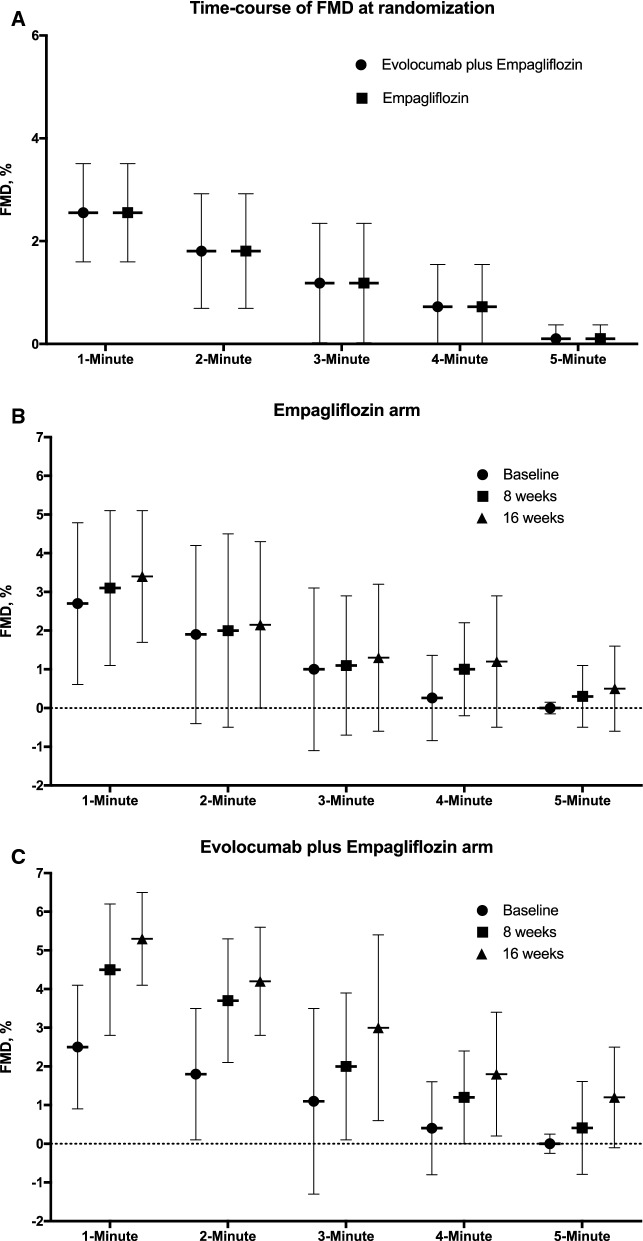
Time course of FMD in patients administered evolocumab plus empagliflozin (circle) or empagliflozin (square). Graph A plots resting FMD curves in both study arms during the pre-randomization phase. Graphs B and C show resting FMD curves for the empagliflozin and evolocumab plus empagliflozin arms, respectively, at baseline, 8 weeks, and after 16 weeks of treatment

Figure 1 shows the time course of FMD from the 1st to the 5th minute after cuff deflation. At randomization, FMD values were comparable between the groups across different time points (Table 1; Fig. 1A). Accordingly, at randomization, the AUC for FMD was similar across treatment groups.

At 8 weeks’ evaluation, the increase in FMD at 1-min was greater in the EE group than in the E group (Table 2). The EE arm also showed a greater increase in FMD at all time points except at 5 min (Table 2). In line with this, there was a higher FMD AUC at 8 weeks in individuals from the EE group when compared to their counterparts.

**Fig. 2:**
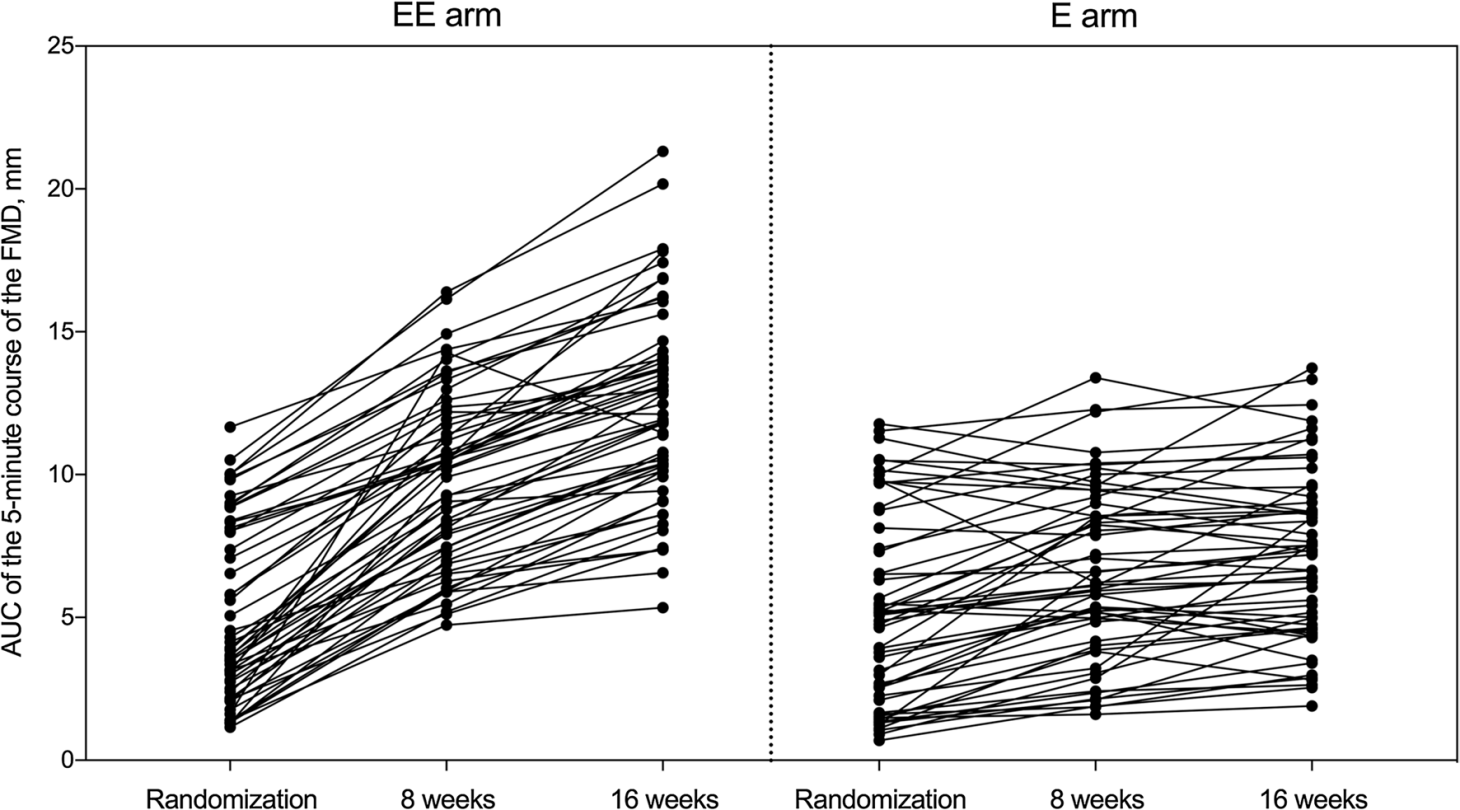
Individual time response curves for the AUC of the 5-min course of the FMD between randomization, 8 weeks, and 16 weeks

At 16 weeks, there was a greater increase of FMD at 1 min in the EE group than in the E group (Table 2). The median increase in FMD was consistently higher in the EE group than that in the E group at all time points (Table 2). While in the EE group 100% of the participants had an increase in both 1-min FMD and AUC for the FMD AUC, in the E group an improvement occurred in 74.5% and 83.6% of the participants, respectively (p < 0.0001). The AUC for FMD at 16 weeks was greater in the EE group than that in the E group (Table 2). There was no interaction between sex or age and the change in FMD at 8 or 16 weeks. Figure 2 shows the individual time response curves for the AUC of the 5-min course of the FMD between randomization, 8 weeks, and 16 weeks.

Figure 1B shows exploratory intragroup analyzes in the empagliflozin group. There were significant changes in the FMD at 1 and 2 min at the 8-week assessment and at all FMD time points at the 16-week assessment (p < 0.05). Figure 1C shows the intragroup analyzes in the evolocumab plus empagliflozin group. There were significant changes at all time points at both the 8- and 16-week assessments (p < 0.01).

### Blood velocity parameters

At randomization, baseline PSV and EDV values at 1-min and 5-min were equivalent in both study arms (Table 1). At 8 weeks, the EE group had a greater increase in EDV at all time points than the E group (Table 2). At the same time point, the EE group had a greater increase in PSV at baseline, but not at 1-min nor 5-min, compared with the E arm (Table 2). At 16 weeks, EDV change from baseline was higher in the EE group than in the E group (Table 2). Similarly, there was a greater increase in PSV at all time points in individuals in the EE group when compared to those in the E group (Table 2).

### NO metabolites

The pretreatment plasma levels of nitrate and nitrite were comparable between the groups at randomization (Table 1). At 8 weeks, post-treatment changes in nitrate levels were similar at baseline, the time point just before the FMD, although a higher increase was reported for the EE group, compared to the E arm, at 1-min and 5-min (Table 2). At 16 weeks, the post-treatment increase in nitrate levels was greater in the EE group than in the E group at all time points (Table 2).

At 8 weeks, the EE group showed a higher median increase in nitrite levels at baseline and at 1-min, but not at 5-min (Table 2). At 16 weeks, the post-treatment increase in nitrite levels was greater in the EE group than in the E group at baseline and 5-min time points. There was a trend toward a greater increase at the 1-min time points, but the difference was not statistically significant (p = 0.058).

### Plasma biomarkers of vascular inflammation and oxidative stress

At randomization, isoprostane, sVCAM-1, and CRP levels were comparable between the groups (Table 1). At 16 weeks, the post-treatment reduction in isoprostane levels was greater in the EE group than in the E. sVCAM1, and CRP levels similarly varied across treatment groups at the 16-week assessment as was for CRP levels at 8 weeks (Table 2).

### Adherence and safety

No serious adverse events were reported in either treatment arm. The adherence to empagliflozin, estimated by the number of pills returned at each visit, was 99.2% and 98.4% in the E and EE groups, respectively. Individuals assigned to the EE group received all anticipated evolocumab doses during the trial. All the COVID-19 exams were negative. During this trial, none of the randomized individuals manifested clinical symptoms suggestive of COVID-19.

## Discussion

Abundant data have shown that evolocumab intensely reduces LDL-C, non-HDL-C, ApoB, Lp(a), and remnant cholesterol in subjects with T2D with and without atherogenic dyslipidemia [17]. In fact, real-life studies, as well as randomized controlled trials, have shown that PCSK9i therapy increases the achievement of goals in individuals with or without T2DM [2, 18–20]. Treatment with evolocumab also reduces the plasma concentration of the most atherogenic form of LDL, the small dense particle, and this reduction is associated with attenuation of carotid stiffness in patients with familial hypercholesterolemia [21]. In clinical trials, evolocumab reduces the risk of cardiovascular events in individuals with atherosclerotic cardiovascular disease or previous manifestation of ischemic stroke [19, 22].

The present study added to this body of evidence that, when compared to the control group, the addition of evolocumab (i) progressively improved FMD at 8 and 16 weeks, (ii) increased the duration of FMD estimated by the AUC of brachial artery diameters at five minutes following cuff deflation at both 8 and 16 weeks of therapy, (iii) increased arterial flow velocity both at peak systole and at end-diastole after 16 weeks of treatment, (iv) increased bioavailability of NO metabolites at 8 and 16 weeks, and (v) reduced oxidative stress estimated by blood isoprostane levels. Furthermore, the study showed that FMD measured after an arterial challenge with a 15-min period of ischemia and reperfusion improved after evolocumab therapy.

### Evolocumab effect on FMD

In recent years, the ability to assess FMD accurately has significantly improved with technological advances and additional biomarker assessments that include NO metabolites. In this scenario, as mentioned above, our laboratory and others have reported improvements in FMD after treatment with SGLT2i [9, 10]. In the present study, we consistently observed improvement in FMD in patients who were randomized to receive empagliflozin without evolocumab in an exploratory intragroup analysis at both 8 and 16 weeks (Fig. 1B). The primary endpoint was set at 16 weeks conservatively; however, at 8 weeks of therapy, the add-on treatment with evolocumab had already demonstrated superiority over the comparative group. These findings were consistent with the observed increase in NO metabolite bioavailability, indicating a contribution from improved endothelial function in increasing FMD. In parallel, the increase in NO bioavailability may have been influenced by a reduction in oxidative stress, as estimated by blood isoprostane levels. In previous trials with statins, these effects on FMD, NO, and oxidative stress markers have been reported in patients with recent acute coronary artery disease, chronic coronary artery disease with and without ventricular dysfunction, chronic inflammatory disease, and T2D [23–27]. In addition, there have been previous reports on the association between the intensity of LDL-C reduction and improvement in FMD in trials of ezetimibe addition or comparison of different statin doses [24, 28–30]. The present study took this body of evidence one step further by showing that the improvement in FMD also occurs after the addition of PCSK9i in T2D patients in use of SGLT2i and on the maximally tolerated dose of statins.

### The duration of arterial dilation after stimulation by the flow

The present study also adds to the current evidence that PCSK9i therapy increases the duration of brachial artery dilation after cuff deflation, evaluated both by the comparison of each time point and by the overall dilation effect estimated by the AUC in the 5 min of the arterial image capture. This effect resulted in the acceleration of arterial flow velocity at both peak systole and end-diastole, effectively showing a reduction in peripheral arterial resistance. This effect was consistent with the increase in NO metabolites 1 and 5 min after cuff deflation. Although there is evidence of direct interaction between PCSK9 and oxidative stress in endothelial cells, the increase in the availability of NO and the consequent increase in FMD is likely to result from the increase in the expression and activity of nitric oxide synthase and the reduction in the production of superoxide anions as a consequence of the decrease in plasma cholesterol-rich lipoprotein concentration [31, 32].

### Endothelial-mediated dilation after ischemia and reperfusion injury

Although endothelial function in baseline conditions is a predictor of cardiovascular events in the long term, in the setting of acute coronary heart disease, ischemia and reperfusion injury can impair endothelial function such that reperfusion is inadequately performed [33]. Thus, the analysis of FMD after a period of ischemia and reperfusion aimed to test the effect of PCSK9i therapy on endothelial functional reserve after I/R injury. The same approach was previously tested in a study that demonstrated the beneficial effects of statin therapy [34]. Consistent with the statin study, we observed progressive improvement in post-I/R injury FMD after 8 and 16 weeks of evolocumab treatment.

### Inflammatory markers’ response to the therapy

We did not find any effect of evolocumab therapy on the markers of vascular and systemic inflammatory activity, namely CRP or sVCAM. It is possible that the patients who were included had low levels of these systemic inflammatory markers at baseline, which may have influenced our results. However, despite the existence of molecular mechanisms indicating the direct participation of PCSK9 in endothelial dysfunction and arterial inflammation, a large set of previous clinical trials have found no change in systemic inflammatory markers after PCSK9i therapy [35, 36]. Although there is no doubt about the early involvement of inflammation in endothelial dysfunction and atherogenesis, only 14% of patients with undisputed vascular inflammation, for example on admission for myocardial infarction, have elevated CRP [37]. This apparent paradox indicates the existence of aspects of vascular inflammation not captured by elevations in CRP [38]. Thus, the lack of change in both inflammatory markers doesn't prove the absence of an anti-inflammatory effect of this therapy.

### Study limitations

The present study has some limitations. The most relevant of these is the fact that we used surrogate endpoints instead of hard endpoints; therefore, the study should not guide clinical decisions. What was intended and demonstrated was that there is plausibility for a beneficial additive effect with treatment with PCSK9i in patients with T2D, even if they are on statins and with optimized medical treatment. Another limitation of the study is its short duration concerning the expected time for the effects of lipid-lowering therapies to take place. However, using FMD as an endpoint allows for shorter trial durations because of the quick changes in the vasomotor aspect of endothelial function and serves as a platform for long-term clinical trials that can be more robustly designed.

### Clinical perspectives

The use of PCSk9i in patients with T2D is effective in reducing the incidence of cardiovascular events [2, 3]. In these studies, new therapies for T2D were not systematically used. Clinical trials have shown that SGLT2i and GLP-1a are effective in reducing cardiovascular events and total mortality in T2D patients [1]. Therefore, the additive value of adding PCSK9i for T2D patients on optimal and current therapy is unknown. In this context, the present study demonstrated that the addition of evolocumab improves baseline endothelial function and even residual endothelial function after I/R injury in the short term (up to eight weeks). Although this study provides a solid basis for plausibility consideration, the clinical implications of these findings will rely on long-term clinical trials designed with clinical endpoints.

## Conclusion

In T2D individuals under treatment with SGLT2i, the addition of evolocumab improves endothelium-mediated arterial dilation and NO production under both resting conditions and after stimulation with I/R. Our results indicate that there is a benefit in the association of these two therapies and provide full plausibility for conducting clinical trials with hard endpoints.

## Supplementary Information

**Additional file 1: Table S1. **Absolute values at each time-point. **Table S2. **Medications in use at randomization. **Figure S1. **Study design. **Figure S2. **FMD Protocol. **Figure S3. **Flow Diagram.

## Data Availability

The BHS group had intellectual property rights to the research data. The data may be made available by the corresponding author upon request.

## Abbreviations

SGLT2i: Sodium-glucose cotransporter 2 inhibitors
T2D: Type 2 diabetes
PCSK9-i: Proprotein convertase subtilisin/kexin 9
E: Empagliflozin
EE: Empagliflozin plus evolocumab
FMD: Flow-mediated dilation
IQR: Interquartile range
GLP-1a: Glucagon-like peptide 1 agonists
EXCEED- BHS3 Trial: EXpanded Combination of Evolocumab plus Empagliflozin on Diabetes Trial
LDL-C: Low-density lipoprotein cholesterol
BP: Blood pressure
ABPM: Ambulatory BP monitoring
AST: Aspartate aminotransferase
ALT: Alanine aminotransferase
CRP: High-sensitive C-reactive protein
VLDL: Very-low-density lipoprotein
HDL: High-density lipoprotein
CKD-EPI: Chronic Kidney Disease Epidemiology Collaboration
sVCAM-1: Soluble vascular cell adhesion molecule 1
AUC: Area under the curve
PSV: Peak systolic velocity
EDV: End-diastolic velocity
SD: Standard deviation
BMI: Body mass index
NO: Nitric oxide
I/R injury: Ischemia and reperfusion injury

## References

[1] Zheng SL, Roddick AJ, Aghar-Jaffar R, Shun-Shin MJ, Francis D, Oliver N, Meeran K (2018). Association between use of sodium-glucose cotransporter 2 inhibitors, glucagon-like peptide 1 agonists, and dipeptidyl peptidase 4 inhibitors with all-cause mortality in patients with type 2 diabetes: a systematic review and meta-analysis. JAMA.

[2] Ray KK, Colhoun HM, Szarek M, Baccara-Dinet M, Bhatt DL, Bittner VA, Budaj AJ, Diaz R, Goodman SG, Hanotin C (2019). Effects of alirocumab on cardiovascular and metabolic outcomes after acute coronary syndrome in patients with or without diabetes: a prespecified analysis of the ODYSSEY OUTCOMES randomised controlled trial. Lancet Diabetes Endocrinol.

[3] Sabatine MS, Leiter LA, Wiviott SD, Giugliano RP, Deedwania P, De Ferrari GM, Murphy SA, Kuder JF, Gouni-Berthold I, Lewis BS (2017). Cardiovascular safety and efficacy of the PCSK9 inhibitor evolocumab in patients with and without diabetes and the effect of evolocumab on glycaemia and risk of new-onset diabetes: a prespecified analysis of the FOURIER andomized controlled trial. Lancet Diabetes Endocrinol.

[4] Deanfield JE, Halcox JP, Rabelink TJ (2007). Endothelial function and dysfunction: testing and clinical relevance. Circulation.

[5] Flammer AJ, Anderson T, Celermajer DS, Creager MA, Deanfield J, Ganz P, Hamburg NM, Luscher TF, Shechter M, Taddei S (2012). The assessment of endothelial function: from research into clinical practice. Circulation.

[6] Shi Y, Vanhoutte PM (2017). Macro- and microvascular endothelial dysfunction in diabetes. J Diabetes.

[7] Broxterman RM, Witman MA, Trinity JD, Groot HJ, Rossman MJ, Park SY, Malenfant S, Gifford JR, Kwon OS, Park SH (2019). Strong relationship between vascular function in the coronary and brachial arteries. Hypertension.

[8] Yeboah J, Folsom AR, Burke GL, Johnson C, Polak JF, Post W, Lima JA, Crouse JR, Herrington DM (2009). Predictive value of brachial flow-mediated dilation for incident cardiovascular events in a population-based study: the multi-ethnic study of atherosclerosis. Circulation.

[9] Batzias K, Antonopoulos AS, Oikonomou E, Siasos G, Bletsa E, Stampouloglou PK, Mistakidi CV, Noutsou M, Katsiki N, Karopoulos P (2018). Effects of newer antidiabetic drugs on endothelial function and arterial stiffness: a systematic review and meta-analysis. J Diabetes Res.

[10] Sposito AC, Breder I, Soares AAS, Kimura-Medorima ST, Munhoz DB, Cintra RMR, Bonilha I, Oliveira DC, Breder JC, Cavalcante P (2021). Dapagliflozin effect on endothelial dysfunction in diabetic patients with atherosclerotic disease: a randomized active-controlled trial. Cardiovasc Diabetol.

[11] Maulucci G, Cipriani F, Russo D, Casavecchia G, Di Staso C, Di Martino L, Ruggiero A, Di Biase M, Brunetti ND (2018). Improved endothelial function after short-term therapy with evolocumab. J Clin Lipidol.

[12] Leucker TM, Gerstenblith G, Schar M, Brown TT, Jones SR, Afework Y, Weiss RG, Hays AG (2020). Evolocumab, a PCSK9-monoclonal antibody, rapidly reverses coronary artery endothelial dysfunction in people living with HIV and people with dyslipidemia. J Am Heart Assoc.

[13] Breder I, Cunha Breder J, Bonilha I, Munhoz DB, Medorima STK, Oliveira DC, do Carmo HR, Moreira C, Kontush A, Zimetti F (2020). Rationale and design of the expanded combination of evolocumab plus empagliflozin in diabetes: EXCEED-BHS3 trial. Ther Adv Chronic Dis.

[14] Thijssen DHJ, Bruno RM, van Mil ACCM, Holder SM, Faita F, Greyling A, Zock PL, Taddei S, Deanfield JE, Luscher T (2019). Expert consensus and evidence-based recommendations for the assessment of flow-mediated dilation in humans. Eur Heart J.

[15] Barroso WKS, Rodrigues CIS, Bortolotto LA, Mota-Gomes MA, Brandão AA, Feitosa ADM, Machado CA, Poli-de-Figueiredo CE, Amodeo C, MionJúnior D (2021). Brazilian guidelines of hypertension—2020. Arq Bras Cardiol.

[16] WHO (2010). Guidelines on drawing blood: best practices in phlebotomy.

[17] Lorenzatti AJ, Monsalvo ML, Lopez JAG, Wang H, Rosenson RS (2021). Effects of evolocumab in individuals with type 2 diabetes with and without atherogenic dyslipidemia: an analysis from BANTING and BERSON. Cardiovasc Diabetol.

[18] Fischer LT, Hochfellner DA, Knoll L, Pottler T, Mader JK, Aberer F (2021). Real-world data on metabolic effects of PCSK9 inhibitors in a tertiary care center in patients with and without diabetes mellitus. Cardiovasc Diabetol.

[19] Sabatine MS, Leiter LA, Wiviott SD, Giugliano RP, Deedwania P, De Ferrari GM, Murphy SA, Kuder JF, Gouni-Berthold I, Lewis BS (2017). Cardiovascular safety and efficacy of the PCSK9 inhibitor evolocumab in patients with and without diabetes and the effect of evolocumab on glycaemia and risk of new-onset diabetes: a prespecified analysis of the FOURIER randomised controlled trial. Lancet Diabetes Endocrinol.

[20] Ray KK, Leiter LA, Muller-Wieland D, Cariou B, Colhoun HM, Henry RR, Tinahones FJ, Bujas-Bobanovic M, Domenger C, Letierce A (2018). Alirocumab vs usual lipid-lowering care as add-on to statin therapy in individuals with type 2 diabetes and mixed dyslipidaemia: the ODYSSEY DM-DYSLIPIDEMIA randomized trial. Diabetes Obes Metab.

[21] Di Minno MND, Gentile M, Di Minno A, Iannuzzo G, Calcaterra I, Buonaiuto A, Di Taranto MD, Giacobbe C, Fortunato G, Rubba POF (2020). Changes in carotid stiffness in patients with familial hypercholesterolemia treated with EvolocumabI: a prospective cohort study. Nutr Metab Cardiovasc Dis.

[22] Gil-Nunez A, Masjuan J, Montaner J, Castellanos M, Segura T, Cardona P, Tembl JI, Purroy F, Arenillas J, Palacio E (2022). Proprotein convertase subtilisin/kexin type 9 inhibitors in secondary prevention of vascular events in patients with stroke: consensus document and practice guidance. Neurologia (Engl Ed).

[23] Ikdahl E, Hisdal J, Rollefstad S, Olsen IC, Kvien TK, Pedersen TR, Semb AG (2015). Rosuvastatin improves endothelial function in patients with inflammatory joint diseases, longitudinal associations with atherosclerosis and arteriosclerosis: results from the RORA-AS statin intervention study. Arthritis Res Ther.

[24] Kawagoe Y, Hattori Y, Nakano A, Aoki C, Tanaka S, Ohta S, Iijima T, Tomizawa A, Jojima T, Kase H (2011). Comparative study between high-dose fluvastatin and low-dose fluvastatin and ezetimibe with regard to the effect on endothelial function in diabetic patients. Endocr J.

[25] Murrow JR, Sher S, Ali S, Uphoff I, Patel R, Porkert M, Le NA, Jones D, Quyyumi AA (2012). The differential effect of statins on oxidative stress and endothelial function: atorvastatin versus pravastatin. J Clin Lipidol.

[26] Oikonomou E, Siasos G, Zaromitidou M, Hatzis G, Mourouzis K, Chrysohoou C, Zisimos K, Mazaris S, Tourikis P, Athanasiou D (2015). Atorvastatin treatment improves endothelial function through endothelial progenitor cells mobilization in ischemic heart failure patients. Atherosclerosis.

[27] Sposito AC, Santos SN, de Faria EC, Abdalla DS, da Silva LP, Soares AA, Japiassu AV, Quinaglia e Silva JC, Ramires JA, Coelho OR (2011). Timing and dose of statin therapy define its impact on inflammatory and endothelial responses during myocardial infarction. Arterioscler Thromb Vasc Biol..

[28] Egede R, Jensen LO, Hansen HS, Antonsen L, Hansen KN, Junker A, Thayssen P (2012). Effect of intensive lipid-lowering treatment compared to moderate lipid-lowering treatment with rosuvastatin on endothelial function in high risk patients. Int J Cardiol.

[29] Nochioka K, Tanaka S, Miura M, do Zhulanqiqige E, Fukumoto Y, Shiba N, Shimokawa H (2012). Ezetimibe improves endothelial function and inhibits Rho-kinase activity associated with inhibition of cholesterol absorption in humans. Circ J.

[30] Westerink J, Deanfield JE, Imholz BP, Spiering W, Basart DC, Coll B, Kastelein JJ, Visseren FL (2013). High-dose statin monotherapy versus low-dose statin/ezetimibe combination on fasting and postprandial lipids and endothelial function in obese patients with the metabolic syndrome: the PANACEA study. Atherosclerosis.

[31] Vogel RA (1999). Cholesterol lowering and endothelial function. Am J Med.

[32] Ragusa R, Basta G, Neglia D, De Caterina R, Del Turco S, Caselli C (2021). PCSK9 and atherosclerosis: looking beyond LDL regulation. Eur J Clin Invest.

[33] Yang Q, He GW, Underwood MJ, Yu CM (2016). Cellular and molecular mechanisms of endothelial ischemia/reperfusion injury: perspectives and implications for postischemic myocardial protection. Am J Transl Res.

[34] Liuni A, Luca MC, Gori T, Parker JD (2010). Rosuvastatin prevents conduit artery endothelial dysfunction induced by ischemia and reperfusion by a cyclooxygenase-2-dependent mechanism. J Am Coll Cardiol.

[35] Sahebkar A, Di Giosia P, Stamerra CA, Grassi D, Pedone C, Ferretti G, Bacchetti T, Ferri C, Giorgini P (2016). Effect of monoclonal antibodies to PCSK9 on high-sensitivity C-reactive protein levels: a meta-analysis of 16 randomized controlled treatment arms. Br J Clin Pharmacol.

[36] Momtazi-Borojeni AA, Sabouri-Rad S, Gotto AM, Pirro M, Banach M, Awan Z, Barreto GE, Sahebkar A (2019). PCSK9 and inflammation: a review of experimental and clinical evidence. Eur Heart J Cardiovasc Pharmacother.

[37] Sposito AC, Alvarenga BF, Alexandre AS, Araujo AL, Santos SN, Andrade JM, Ramires JA, Quinaglia ESJC, Coelho OR (2011). Most of the patients presenting myocardial infarction would not be eligible for intensive lipid-lowering based on clinical algorithms or plasma C-reactive protein. Atherosclerosis..

[38] Soehnlein O, Libby P (2021). Targeting inflammation in atherosclerosis—from experimental insights to the clinic. Nat Rev Drug Discov.

