# Supplementary material for: Evolocumab on top of empagliflozin improves endothelial function of individuals with diabetes: randomized active-controlled trial
Source: Cardiovasc Diabetol. 2022 Aug 6;21:147. doi: 10.1186/s12933-022-01584-8 (PMC9356512; doi:10.1186/s12933-022-01584-8)
Supplement: Supplementary file 1 — Additional file 1: Table S1. Absolute values at each time-point. Table S2. Medications in use at randomization. Figure S1. Study design. Figure S2. FMD Protocol. Figure S3. Flow Diagram. [file 12933_2022_1584_MOESM1_ESM.docx]

**ADDITIONAL MATERIAL**

**Evolocumab on top of empagliflozin improves endothelial function of individuals with diabetes: randomized active-controlled trial**

**Table S1. Absolute values at each time-point**

|  | **Time-point** | | | | | |
| --- | --- | --- | --- | --- | --- | --- |
|  | **8 weeks** | | | **16 weeks** | | |
| **Variable** | **EE** | **E** | **p-value** | **EE** | **E** | **p-value** |
| Heart rate, bpm | 80±8.6 | 81±11 | 0.723 | 70±11 | 72±10 | *0.490* |
| Office SBP, mmHg | 129±13 | 129±14 | 0.923 | 126±14 | 129±15 | *0.332* |
| Office DBP, mmHg | 78±9.2 | 79±9.4 | 0.408 | 78±8.8 | 80±9.1 | *0.172* |
| Body weight, Kg | 86±16 | 86±15 | 0.934 | 85±16 | 85±15 | *0.900* |
| Height, m | 1.7±0.1 | 1.7±0.1 | 0.848 | 1.7±0.1 | 1.7±0.1 | *0.846* |
| Body Mass Index, Kg/m^2^ | 29±5.1 | 30±4.1 | 0.846 | 30±4.9 | 30±4.2 | *0.809* |
| Waist circumference, cm | 104±13 | 103±11 | 0.742 | 103±13 | 104±10 | *0.794* |
| ***Biochemical analysis*** | | | | | | |
| Hemoglobin, g/dL | 14±1.4 | 13±1.6 | 0.754 | 14±2.1 | 14±1.6 | *0.234* |
| Fasting Blood Glucose, mg/dL | 134 (30) | 123 (34) | 0.175 | 137 (33) | 127 (32) | *0.464* |
| HbA1c, % | 7.0 (0.8) | 6.9 (0.8) | 0.832 | 7.1 (0.7) | 6.9 (0.7) | *0.512* |
| LDL-C, mg/dL | 29 (14) | 68 (12) | < 0.001 | 23 (13) | 69 (22) | *< 0.001* |
| HDL-C, mg/dL | 41 (12) | 39 (13) | 0.153 | 42 (12) | 41 (17) | *0.521* |
| VLDL-C, mg/dL | 24 (18) | 27 (14) | 0.045 | 24 (15) | 26 (15) | *0.059* |
| Triglycerides, mg/dL | 120 (90) | 135 (67) | 0.036 | 119 (85) | 127 (68) | *0.080* |
| Creatinine, mg/dL | 0.8 (0.3) | 0.9 (0.3) | 0.985 | 0.9 (0.3) | 0.8 (0.3) | *0.486* |
| Urea | 32 (12) | 36 (14) | 0.835 | 36 (12) | 35 (11) | *0.759* |
| C-Reactive Protein, mg/dL | 0.2 (0.4) | 0.2 (0.3) | 0.779 | 0.2 (0.4) | 0.2 (0.3) | *0.813* |
| AST, U/L | 19 (8) | 19 (11) | 0.172 | 17 (8) | 18 (8) | *0.325* |
| ALT, U/L | 23 (10) | 22 (17) | 0.202 | 20 (12) | 20 (14) | *0.364* |
| ***24h BP Monitoring*** |  |  |  |  |  |  |
| SBP, mmHg | na | na | na | 116±12 | 118±13 | *0.295* |
| DBP, mmHg | na | na | na | 70±8.1 | 71±8.1 | *.0.438* |

Systolic blood pressure; DBP : Diastolic blood pressure; HbA1c : glycosylated hemoglobin; LDL-C : low-density lipoprotein cholesterol; HDL-C : high-density lipoprotein cholesterol; VLDL : very low-density lipoprotein cholesterol; AST : Aspartate transaminase; ALT : Alanine transaminase; na : not available

**Table S2. Medications in use at randomization**

| **Variable** | **E** | **EE** | ***p-value*** |
| --- | --- | --- | --- |
| Angiotensin receptor blockers, n (%) | 54 (98) | 54 (98) | *0,99* |
| Calcium channel blocker, n (%) | 14 (25) | 6 (11) | *0.04* |
| Diuretics, n (%) | 18 (33) | 21 (38) | *0.69* |
| Beta blocker, n (%) | 14 (25) | 15 (27) | *0.99* |
| Oral hypoglycemic, n (%) | 24 (44) | 21 (38) | *0.69* |
| Sulfonylureas, n (%) | 24 (44) | 25 (45) | *0.99* |
| Statin use, % | 100 | 100 | *1.00* |
| Rosuvastatin, mg (n) | 20 ± 12 (18) | 18 ± 11 (21) | *0.41* |
| Simvastatin, mg (n) | 19 ± 11 (37) | 26 ± 12 (34) | *0.69* |
| High-dose statin, n (%) | 27 (50) | 29 (54) | *0.89* |

High-dose statin: Rosuvastatin 20/40mg, Simvastatin 40mg

**Figure S1. Study design**

**
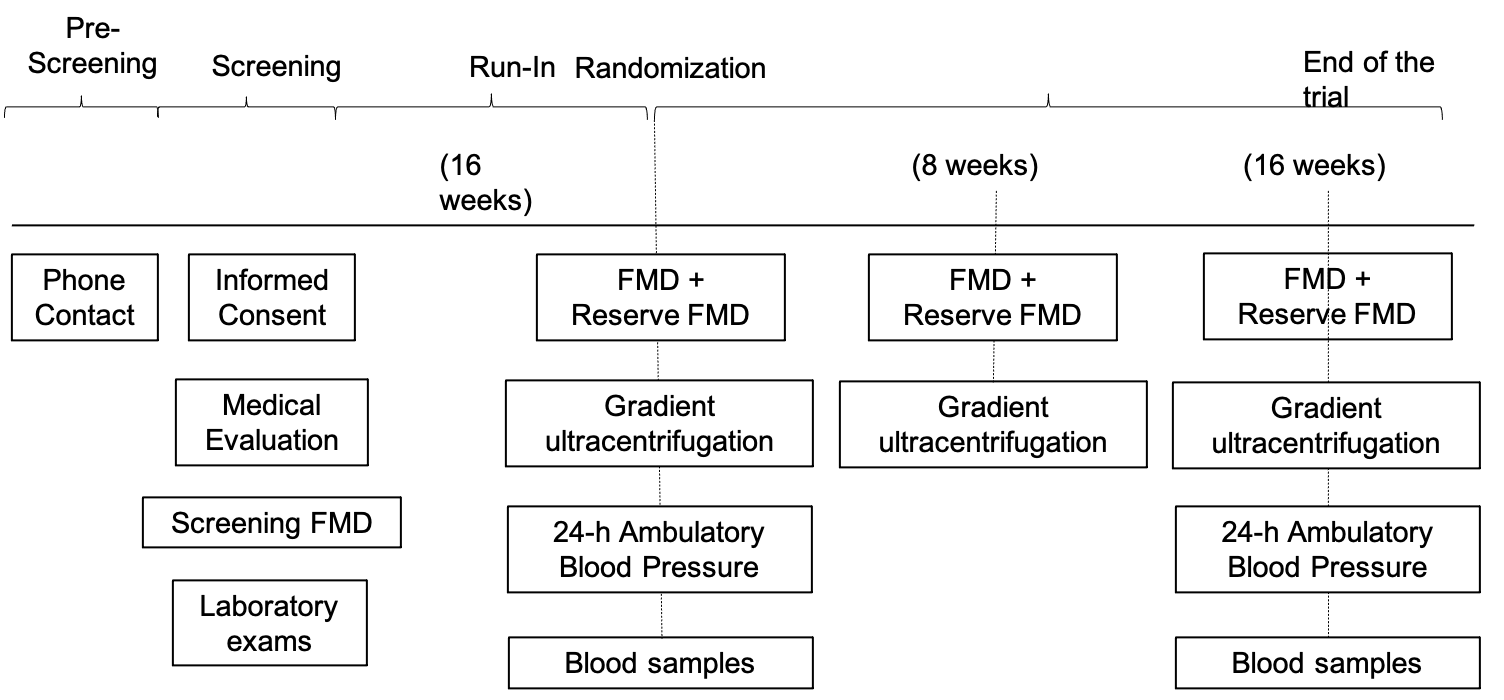
**

**Figure S2. FMD Protocol**

**
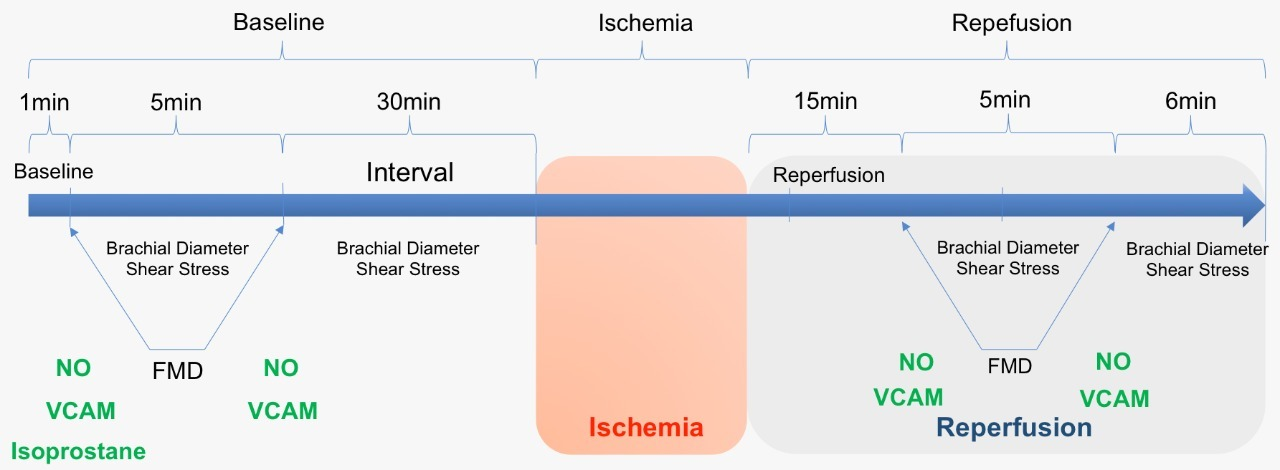
**

**Figure S3. Flow Diagram**
